# How oncology teams can be patient‐centred? opportunities for theoretical improvement through an empirical examination

**DOI:** 10.1111/hex.12847

**Published:** 2018-11-08

**Authors:** Karine Bilodeau, Dominique Tremblay

**Affiliations:** ^1^ Faculty of Nursing University of Montreal, Montreal QC Canada; ^2^ Faculty of Medicine and Health Sciences University of Sherbrooke Longueuil QC Canada; ^3^ Hôpital Charles‐LeMoyne Research Center Longueuil Quebec Canada

**Keywords:** cancer, framework, interprofessional practice, multidisciplinary team, patient‐centred, teamwork

## Abstract

**Background:**

In the context of interprofessional practice, a patient‐centred approach is recommended, which generally means power‐sharing, shared decision making and involving patients as part of the health‐care team. These aspects, which are essential to “patient‐centred” practice, do not appear to be sufficient to illustrate the full richness of this practice.

**Objective:**

This article aimed to understand how interprofessional patient‐centred (IPPC) practice in oncology teams contributes to creating a more positive experience for patients. Objectives were to (a) describe the IPPC practice of oncology teams using the IPPC Practice Framework; (b) determine the usefulness of this framework; and (c) offer alternative proposals for expanding our understanding of IPPC practice.

**Design:**

A secondary analysis was performed with data from a multicase study designed to explore the effects of interdisciplinary work among oncology teams. Data were provided from six focus groups with professionals (n = 22) and patients diagnosed with cancer (n = 16). An iterative content analysis was performed.

**Results:**

Applying the theoretical framework to data analysis enabled us to distinguish between the IPPC practice of the different teams and structure the data collected in order to show the processes and place them in context. However, it proved to be difficult to describe the central component of the theoretical framework, *patient‐centred processes*. This situation raises new hypotheses for representing practice in a real‐life context. An alternative perspective for illustrating IPPC practice is therefore proposed.

**Conclusion:**

This study emphasizes the importance of exploring the utility of theoretical frameworks and refining them in order to broaden our understanding of IPPC practice.

## 1 BACKGROUND

Over the past twenty plus years, many initiatives have emerged that focus on the necessity of working in interprofessional teams. Specifically, recommendations have been made on interprofessional patient‐centred practice, with reference to power‐sharing, shared decision making and giving patients an active role in the health‐care team.^1^, ^2^, ^3^, ^4^, ^5^ While the latter aspects are essential to “patient‐centred” practice,^6^, ^7^ they do not represent the totality of this practice. A “patient‐centred” approach must also address the relationship between the patient and health‐care professionals, as well as recognizing the uniqueness of the individual.^6^, ^7^, ^8^, ^9^, ^10^, ^11^ More recently, a Canadian report defines that this approach enables patients diagnosed with cancer to be more engaged in their care, helps to define more appreciate health‐care services and improves patients experience.^12^ To our knowledge, only one study has documented an interprofessional practice that is patient‐centred based on the shared perspectives of cancer patients, their families and health‐care professionals in oncology.^13^ What emerges is that the desired practice should consist of the patient's engagement (at her own pace), not imposing professional values, and consistent collaboration among all members of the team, including the patient. However, it has been well documented that the context of interprofessional practice can compromise patient‐centred care.^14^, ^15^ Looking at the evidence reminds us of the importance of considering a patient‐centred approach in interprofessional practice, and also of being able to recognize such an approach in clinical practice.

To fully grasp the concept of interprofessional patient‐centred practice, it is useful to identify promising theoretical frameworks that could be used in studies on the topic. Usually, theoretical frameworks are used to explain study objectives by presuming correlations among key factors, variables, or theoretical constructs.^16^ They can also guide the ways in which data are collected, described and interpreted,^17^ presenting a cartography of the topic to be studied. The interprofessional patient‐centred (IPPC) Practice Framework illustrates this type of practice and seems useful to an oncology context^18^ because it proposes a promising point of view. Indeed, this framework proposes to explain the IPPC practice, a patient‐centred process that includes working with patients’ needs, being involved, having an empathetic presence, sharing decision making and offering holistic care. This process seems useful, but we do not know whether it is appropriate for explaining the IPPC practice properly in oncology settings.

This article aimed to understand how interprofessional patient‐centred practice (IPPC) in oncology teams contributes to creating a more positive experience for patients. First, we try to explain the IPPC practice of three oncology teams using the IPPC Practice Framework. Second, we determine the usefulness of this emergent framework to explain the IPPC practice in real context. Third, through a back‐and‐forth process, new proposals will be offered to expand our understanding of this practice.

A further note: In the manuscript, we use the appellation “patient‐centred” because it is worldwide used in textbook, scientific literature and indexed keywords. We acknowledge that “patient‐centred” appellation can seem restrictive to understand how an interprofessional team can provide personalized care. Some authors and organizations prefer to use the appellation “person‐centred” to recognize the human being behind the term “patient”.^19^, ^20^

### 1.1 An interprofessional adaptation of the *Person‐Centred Practice Framework*

During her doctoral studies, Bilodeau adapted the *Person‐Centred Practice Framework* developed by McCormack and McCance^20^ to the context of interprofessional teams in oncology. The authors refer to a “person‐centred” practice for emphasizing the humanhood of each person. The adapted theoretical framework, the *IPPC Practice Framework*, provided a theoretical perspective for a constructivist study aiming to describe the IPPC practice of oncology teams.^21^ It guided the ontological and epistemological positioning of the study and the development of data collection tools (interview guides, observation grid). Further reflection raised the question of the *IPPC Practice Framework*'s potential for guiding the analysis of data around a theme and a related context, that is, patient‐centred care provided by an interprofessional oncology team.

The original theoretical framework, the *Person‐Centred Nursing Framework,* 20, 22 was the result of empirical work on patient‐centred practice in geriatrics^23^ and the experience of *caring* in nursing.^24^ The objective was to spell out what person‐centred nursing care involves. This theoretical framework includes four theoretical constructs: *prerequisites, care environment, person‐centred processes* and *outcomes*. The *Person‐Centred Nursing Framework* was validated in studies conducted in geriatrics and acute‐care settings. Although the authors have modified their theoretical framework over the years for the interprofessional context,^25^ the concept of teamwork is still underrepresented. It was for that reason that the original theoretical framework of McCormark and McCance^20^ was adapted to the interprofessional oncology context.^18^, ^21^ The main adaptations were the use of “patient‐centred” appellation and the addition of *interprofessional meeting space*, inspired by Couturier's work on interdisciplinarity in primary care nursing and social work.^26^, ^27^, ^28^ Couturier explained that “inter‐” means a space of encounters, characterized by movement. Thus, the *interprofessional meeting space* 28 is where the IPPC practice is carried out in a space for mutual exchanges between professionals and patients. Three of the four original constructs were then modified to emphasize teamwork (Table 1). The adaptation also integrated the patient into various theoretical constructs. According to the *IPPC Practice Framework (adapted version),* it is in an *interprofessional meeting space* that the team, possessing the *prerequisites,* should be able to deal with a *care environment* to fully perform a *patient‐centred process* that may result in *outcomes*.

**Table 1:** Interprofessional patient‐centred (IPPC) Practice Framework components^18^ adapted from McCormack et al^20^

***Interprofessional meeting space*** a means meetings that enable professionals to share among themselves, demonstrate creativity and transform their practice. A planned interprofessional intervention should develop in this way. ***Prerequisites*** a places the emphasis on the team's attributes, which include communication between team members and the patient, establishing common values between team members and the patient, and quality of teamwork (clear objectives, active participation, engagement, support for innovations). ***Care environment*** places the emphasis on the context in which care is provided and features: a care system that facilitates shared decision making, a good relationship among team members, support for the organizational system and power‐sharing between various professionals and the patient^a^. ***Patient‐centred processes*** places the emphasis on care provided in the course of various activities of the team, including working with needs and wants of the patient, be engaged in patient care, having an empathetic presence with the patient^b^, sharing decision making with the patient and offering holistic care to the patient. ***Outcomes*** is the central component, meaning the anticipated results of effective *patient‐centred processes* based on the following themes: satisfaction with care, participation in care, a feeling of well‐being and creating a therapeutic environment.

a Addition

b Adaptation

## 2 METHOD

The *IPPC Practice Framework* was used as a guide for the secondary analysis of data from a study by Tremblay et al^29^ We decided to perform a secondary analysis given preliminary results that highlighted the association between patient‐centred care and interprofessional teamwork. Secondary analysis involves conducting a new study using data from previous studies (Heaton, 2008). The data are used to answer different questions from the primary study. This method is useful when it is possible to have access to raw data from the primary study, when data from the primary study are compatible with the new research question, and when coded documents from the primary study are still available (Heaton, 2008).

### 2.1 Description of the primary study

The data come from a multicase study that involved a realistic evaluation^30^ of conditions for the production of interdisciplinary teamwork outcomes in oncology teams.^29^ Based on the effects of different levels of interdisciplinarity (high vs low), the primary study was designed to explain the mechanisms involved in producing interdisciplinary teamwork outcomes in a given context. One of the effects in the study was patient‐centred care.^31^ The cases (n = 7) represented oncology teams made up of a range of professionals, for example, oncologist, nurse, pharmacist, social worker, psychologist and nutritionist. The study was approved by the Research Ethics Board of the Charles‐LeMoyne Hospital Research Centre (ref. number MP‐HCLM‐13‐034) and was valid to cover the use of the data for the secondary analysis.

### 2.2 Sources for the secondary analysis

Data from the secondary analysis provided by three teams (cases) chosen for their differences in mission, academic affiliation, level of interdisciplinary and size, as well as their diversity and geographic locations, were used (Table 2). This choice makes it possible to represent the diversity of clinical practice in an oncology setting, with a total of six homogeneous focus groups of professionals (n = 22) and patients diagnosed with cancer (n = 16). In focus groups, members of the interprofessional teams were invited to discuss their perception of the effects of an interdisciplinary work on the care experience. For the focus group of patients diagnosed with cancer, a vignette describing the story of a patient using oncology health‐care services was used. Participants were invited to give their explanations of the effects of care, notably patient‐centred care provided by an oncology team. The focus group discussions were recorded and then transcribed verbatim.

**Table 2:** Characteristics of teams

Characteristics	Team #1	Team #2	Team #3
Number participants			
Patients with cancer	8	9	5
Professionals	6	6	4
Mission	Regional	Regional	Local
Academic affiliation	√		√
Level of interdisciplinarity	Intermediate	High	High
Team size and diversity^a^	Small	Large	Small
Location	Rural	Semi‐rural	Urban

a ≤8 different types of professionals = small; >8 = large.

### 2.3 Analysis

A content analysis was performed on the data, consisting of an iterative process that included the following activities: condensation, presentation of data, and elaboration and verification of conclusions.^16^ An initial coding grid was created based on the five components of the *IPPC Practice Framework*. A content analysis was performed for each case based on two coding cycles. The first coding cycle, based on a procedural method,^32^ was designed to attribute specific codes linked to the theoretical framework of sections of the transcriptions. In the second cycle, content‐analysis summary tables were put together to link the dimensions of the *IPPC Practice Framework* for each team. Then, a transverse analysis was performed to elicit similarities and differences between cases. To validate an unexpected conclusion during the writing of this manuscript, a third cycle of coding was done. Data were set to produce an alternative illustration of emergent findings presented in the discussion section. QDA Miner v.4.0.11 software was used to manage the qualitative data.^33^ Credibility (internal validity) was ensured by listening to all the focus groups and consulting the field notes from the primary study.^33^ The preliminary results were validated with members of the initial research team to make sure that the results made sense in the initial context of the study. For transferability (external validity), a detailed description of the diverse contexts of the teams was put together so that others (patients, researchers, professionals) would be able to identify similarities with their contexts and assess the advance conclusions.^33^

## 3 RESULTS

The interprofessional practice of the three teams was described according to the theoretical framework, in terms of *interprofessional meeting space*,* prerequisites*,* care environment*,* patient‐centred processes* and *outcomes*. Examples were presented to illustrate various aspects of the components of the *IPPC Practice Framework* as described by patients and oncology professionals (Table 3).

**Table 3:** Description of IPPC practice of three oncology teams

IPPC practice components	Team #1	Team #2	Team #3
Interprofessional meeting space Moment of contact with other professionals Interprofessional planned intervention (shared objectives)	Unsatisfactory multidisciplinary meetings. “I took the trouble to go but we left after 20 minutes without having discussed any cases. So why was I there? For nothing!” (Prof #1) Discussions in the hallways. “It's unidisciplinarity when you go and get expertise from one person at a time in the hallway.” (Prof #1) Joint interventions can be difficult to organize because it's hard to reach certain professionals. “There's no doctor available so we run around to find someone else.” (Prof #1)	Poor attendance at multidisciplinary meetings. “We have time slots set up, every Thursday we send a notice of meeting and ask ‘Do you have cases to present to the inter team?’ but often no one has any cases to present.” (Prof #4) Discussions on the phone or on the ward. “So I'll call the person, I think that settles things…it's often like that.” (Prof #5) Team's ability to complete joint projects.	Poor attendance at multidisciplinary meetings. “I think it's so easy to skip that meeting, there's no value added there.” (Prof #8) Unsatisfactory multidisciplinary meetings. Discussions in the hallways. “We finalize a lot more, make things concrete more than we want to in the hallway, then we go and see the doctor… (…)” (Prof #9) Trouble with formalizing interventions. “What's missing from the official meeting is being able to formalize things. We miss having a doctor present.” (Prof #8) Team's ability to complete joint projects. “You know, that team works really well together. They have a goal.” (Patient #3a)
Prerequisites Communication between team members Establishment of common values Quality of teamwork (clear objectives, participation, involvement, support for innovations)	Less‐than‐optimal communication between team members and between team and patients. Trouble achieving common objectives. “(…) one of our problems is that all the professionals keep their own files (…) I can't read notes made by all the professionals to get an overview.” (Prof #2) There is a vision of treatment‐centred care. “They focus on treatment, treatment – yes, but that's not the only thing.” (Prof #3)	There is communication between team members. Less‐than‐optimal communication between team members and patients. “Yeah, but that's not what we want [a pamphlet]. We want human contact. We want someone to come talk to us. That's what we want. It's not that hard.” (Patient #2a) Interprofessional vision is “not anchored.”	There is communication between team members and between team members and patients. “(…) when I meet a patient, I say ‘how are you? So‐and‐so told me (…) how you're managing this?’ So it's clear that we show the patient that there is communication.” (Prof #8) Harmonious discourse on the team. “I think everyone is on the same wavelength here (Patient #3a) There is some divergence re the vision of continuity of care.
Care environment Shared decision making system Good relationships between team members Supportive organizational system Power‐sharing between professionals and patient Physical environment	Limited power‐sharing between team members. “I've been cut off because I might have had something to say, but it was over. The person said what she had to say, then that was it.” (Prof #3) Pressure from the organization on team performance. “(…) when you think about it, the time I take to go and talk to the pivot nurse, with the doctor, isn't in the statistics, so there's pressure at a given time…”(Prof #2)	Power often attributed to just one discipline. “It's really the doctor's word, really.” (Prof #6)	Team has limited capacity for legitimizing power‐sharing. “Our hands are tied, I can't do a direct consultation so we sneak it through by the patient so he can ask for palliative care (Prof #9) Visible support from the organization. “This isn't just luck, it's not just because there are good people here, management is making an effort.” (Patient #3b)
Patient‐centred processes working with patient's beliefs and values engagement having empathetic presence sharing decision making providing holistic care	Patients given documentation for decision making. “When you meet the specialist you start getting documentation (…) So you know what you have to think about, it's relevant. You have a choice to make, 2 or 3 choices.” (Patient #1a) Recognizing the uniqueness of the individual. “The patient needs to be seen fully in every aspect of his or her suffering.” (Prof #4) Physical care prioritized. “I was happy when the professional told me I didn't look sick (…), but that situation doesn't generate people who take care to see how you're doing.” (Patient #1b)	Professionals encourage patients to make decisions, but patients are surprised by that approach. “How come I'm forced to choose? Well, because it's you, your body, you have to choose. But I have lots of people who are surprised that they need to choose their own treatment.” (Prof #7) Recognizing the uniqueness of the individual. “So you also adapt to the person in front of you because sometimes based on reality you need to spend more time on one part than another as needed.” (Prof #7*)*	Paternalistic attitude of some professionals when it comes to decision making. “(…) people who are still in the old school, so to speak” (Prof #8) Recognizing the uniqueness of the individual. “We don't just evaluate a breast or a spleen or a lung, we also see the whole person so we put professionals in place.” (Prof #9)
Outcomes satisfaction with care involvement in care feeling of well‐being therapeutic environment	Little participation by patient. Mixed satisfaction with care (information, access, continuity of care and services) “Mr. X needs something particular but doesn't know who to call. He needed to take a pamphlet.”(Patient #1c)	Mixed satisfaction with care (information, access, continuity of care and services). “If she reaches you the same day you're lucky (…) Unless it's an emergency, but for information, no. She's really nice, she does good work, but in my experience, that's average.” (Patient #2b)	Patient participation. “I think it's important to be involved because that helped me get through, not just with the staff, but with the other patients.” (Patient #3c) High satisfaction with care. “It met my expectations.” (Patient #3b)

### 3.1 Interprofessional meeting space

For the three teams, the *moment of contact* typically involved opportunistic interactions among the professionals when they met in the hallway or on the ward. The goal of these interactions was to exchange and agree upon interventions with patients in complex or urgent situations. Although interprofessional meetings were held, the professionals of all teams saw no added value in participating, since the exchanges on patients’ situations were quick and brief. Professionals on team #2 stated that although it was a good idea to hold the meeting, it was too bad that meetings were not held regularly. All the teams said that *interprofessional planned interventions* were difficult to organize due to the time it could take to reach professionals, the absence of doctors and their lack of participation, and the lack of coordination between individual interventions by team members. The professionals on team #3 were the only ones who mentioned that projects with team members in the last few years had made it possible to consolidate teamwork and adjust the services offered to patients.

### 3.2 Prerequisites

*Communication between team members and patients* was a major issue for team #1. The professionals explained that team members wrote their evaluations in parallel files, which meant limited capacity for intervention. In addition, the professionals deplored the lack of structure and clear roles within the team. The professionals on team #2 agreed that colleagues were available, polite and receptive to everyone's requests. In terms of communication between team members and patients, the professionals explained that they encouraged patients to use their oncology passport (information pamphlet for patients) to get information. However, the patients did not appreciate the written information in the pamphlet and preferred having contact with professionals to answer their questions. Team #3 stated that there was communication between team members and between the team and patients. The professionals used an orientation session for patients who were starting chemotherapy treatments to present the team and the services available. They also tried to explain to patients that the team discussed their situations as a group. As for *common values*, a common vision did exist within team #3, and the patients noticed that. The situation was different on teams #1 and #2, with professionals expressing the difficulty of preserving *common values*.

### 3.3 Care environment

Looking at the elements in this component, *power‐sharing between professionals and patients* was limited for professionals on the team. There was a medical hierarchy, and the situation limited power‐sharing between professionals, as well as each professional's optimal exercise in their respective fields of practice. *Shared decision making* remained quite difficult to achieve due to the presence of this hierarchy. For example, professionals mentioned that patients preferred decisions to be made by the doctor. Despite the difficulties reported by the professionals, none of the patients on the three teams had noticed these aspects. They said they would rather feel welcomed by their respective teams. As for the *supportive organizational system*, the professionals on team #1 reported that they felt pressured when it came to team performance. They were asked to list their activities. They felt that the requested data were not helpful in presenting a useful picture of interprofessional work. The situation was different for team #3, with patients commenting that the organization offered support for the team.

### 3.4 Patient‐centred processes

For all three teams, *working with patients’ beliefs and values* was described by participants as respecting patients’ wishes and their individual pace, as well as recognizing their uniqueness. However, the patients and professionals on team #1 reported that evaluation tended to focus more on physical symptoms rather than performing a broader assessment. *Shared decision making* differed from team to team. Patients on team #1 noted that it was at the time of their cancer diagnosis that they felt they had a choice to make, which they appreciated. The professionals on team #2 made efforts to encourage decision making, but the patients were still surprised at having a choice to make. The professionals on team #3 noted that some colleagues had difficulty sharing decision making with the patient.

### 3.5 Outcomes

The outcomes show that *satisfaction with care* differed from team to team. The professionals on teams #1 and #2 said that patients were satisfied with services and they felt secure. However, the patients reported some dissatisfaction with the information they were offered, gaining access to professionals on the team and continuity of care. The patients on team #3 were generally satisfied with their care, noting that they had been taken into care and the treatments were in line with their expectations. As for *involvement in care*, the professionals on team #2 commented that patients participated very little in their care, despite their efforts to get them more involved.

## 4 DISCUSSION

The results of this secondary analysis show how the *IPPC Practice Framework* contributes to understanding the connections between teamwork and patient‐centred care in oncology. First of all, by using the theoretical framework, we can see small differences in interprofessional practice among the three teams. However, it was difficult to describe the connections between the context, processes and outcomes to gauge the full complexity of the phenomenon we were studying. We had a hard time with trying to describe the overall patient‐centred processes in explicit terms. Subsequent sections will address the usefulness and limitations of applying the *IPPC Practice Framework* in its current form.

The application of the *IPPC Practice Framework* to data analysis made it possible to detail and distinguish between the interprofessional practice of the teams. For the *prerequisites*, the results suggest problems with communications between team members and patients, as well as the accomplishment of objectives within some teams. As for the *care environment*, the presence of a medical hierarchy hampers shared decision making for both professionals and patients. For the *interprofessional meeting space*, teams reported having planned contact times (meetings), but the meetings were described as unsatisfactory. Based on the results, more informal contacts (eg, discussions in the hallways) seem to be more fruitful. Note that the latter results are consistent with results from several studies documenting the relevance of formal and informal contacts in consolidating teamwork and planning interventions.^13^, ^34^, ^35^, ^36^, ^37^ In addition, although some *outcomes* were documented, it was possible to pinpoint *satisfaction with care*. Patients expressed their appreciation for the information they received, access to services and continuity of care and services. These results are relevant because they spell out patients’ expectations in terms of care and services.^38^, ^39^ The application of the *IPPC Practice Framework* helps to document some elements of the teams’ context and detail what patients expect from their oncology teams.

Although a description of the context for the teams in the study was put together, it was sometimes difficult to see which results were describing *interprofessional meeting space* and which were describing *prerequisites*, as there were similarities between the two components. For the interprofessional meeting space, it was more complicated to describe how the team was able to determine *shared objectives* during *interprofessional planned interventions*. These aspects were not very different from the aspects of the *prerequisites* where the issue was the quality of teamwork, characterized by clear objectives, participation of members, engagement and support for innovation. These two components also seem to be quite close conceptually, limiting the use of the *IPPC Practice Framework* for data analysis. The relevance of the two components in their current forms should therefore be reassessed.

A superficial description of *patient‐centred processes* on the three teams was put together. The situation is deceptive, however, since this description was to have been the original contribution of the application of the *IPPC Practice Framework* to the data analysis. The only data that emerged were some data relating to professionals’ desire to *provide holistic care*, propose that d*ecision making be shared* and *recognize the uniqueness of the individual*. These themes were approached in a general way, without describing how a team managed to complete a *patient‐centred process*. In addition, some aspects of *patient‐centred processes* could not be documented, such as *be engaged in patient care* and *having an empathetic presence with the patient*. One may well wonder why the application of the *IPPC Practice Framework* to the analysis did not help with describing *patient‐centred processes*. This is surprising, given that the data in the primary study stressed the importance of patient‐centred care.^40^ An initial explanation could perhaps be that the teams were unable to complete a *patient‐centred process*. The *IPPC Practice Framework* explains that this process is interlinked with the presence of prerequisites and the effect of the environment. The results obtained lead us to believe that some teams do not have enough prerequisites (eg, communication, common values, quality of teamwork) to be able to accommodate their environment (eg, power‐sharing between professionals and patients). In fact, problems with communications between team members and patients, as well as the accomplishment of joint objectives, were noted. The care environment was also described as not being conducive to power‐sharing within the team. This situation could explain the difficultly of clearly documenting *patient‐centred processes*. That hypothesis is consistent with the work of Salas, Shuffler, Thayer, Bedwell and Lazzara^41^ noting that the makeup of the team, the context in which the team interacts and the culture of the organization, the team and individuals can undermine the quality of teamwork. The theoretical framework may be sufficient to guide the description of the interprofessional context and help to establish whether or not a team is performing *patient‐centred processes*.

A second hypothesis regarding the difficulty of documenting *patient‐centred processes* is linked to the theoretical foundations of the *IPPC Practice Framework*. The original *Person‐centred Practice Framework* was based on humanist and existentialist theoretical perspectives recognizing the dignity of human beings. These perspectives emphasized the importance of the relationship between the professional and the patient. The activities suggested in the *patient‐centred processes* component, such as having an empathetic presence or being fully engaged, are more closely identified with individual rather than interprofessional practice. The results obtained reflect the fact that more activities related to *working with patients’ beliefs and values* and *sharing decision making* appear to have been carried out. Thus, these results allow us to again question the accuracy of the linkages between concepts of patient‐centred processes as presented by the IPCC Practice Framework. Bear in mind that what makes a theoretical framework useful is the way it helps researchers to represent reality. The difficulty of documenting *patient‐centred processes* suggests that the theoretical framework, in its current form, may not be the best way to explain specifically the *patient‐centred processes* performed by the teams.

### 4.1 An unexpected conclusion: an alternative perspective of IPPC practice

In the light of the results obtained, there seems to be a nuance between a *patient‐centred process* seen from an individual perspective and what constitutes a *patient‐centred process* from the team's perspective. During the back‐and‐forth process of analysis, authors were disappointed by the difficulty of properly describing a patient‐centred process. As an objective of this paper, new proposals are offered to expand our understanding of this practice. Looking at the literature, we are reminded that patient‐centred practice includes an interpersonal process and a relationship that changes as the professional and patient come to share a sense of mutual confidence.^7^ Teamwork is described as a process that includes a planning phase and an action phase, interacting cyclically.^42^ The planning phase typically includes planning and evaluating the intervention. The action phase then proposes that each professional on the team complete his or her assigned task, with the team monitoring progress, coordinating activities and supporting members. The latter representation is interesting, as it shows that individual and team practice are intertwined. The collective (patient‐team) and individual (patient‐professional) relationships appear to be complementary and interrelated. This hypothesis calls into question the representation of interprofessional patient‐centred practice as proposed by the *IPPC Practice Framework*. For these reasons, we tested an alternative conclusion for better understand how an oncology team can be patient‐centred within a third cycle of data coding.

**Figure 1:**
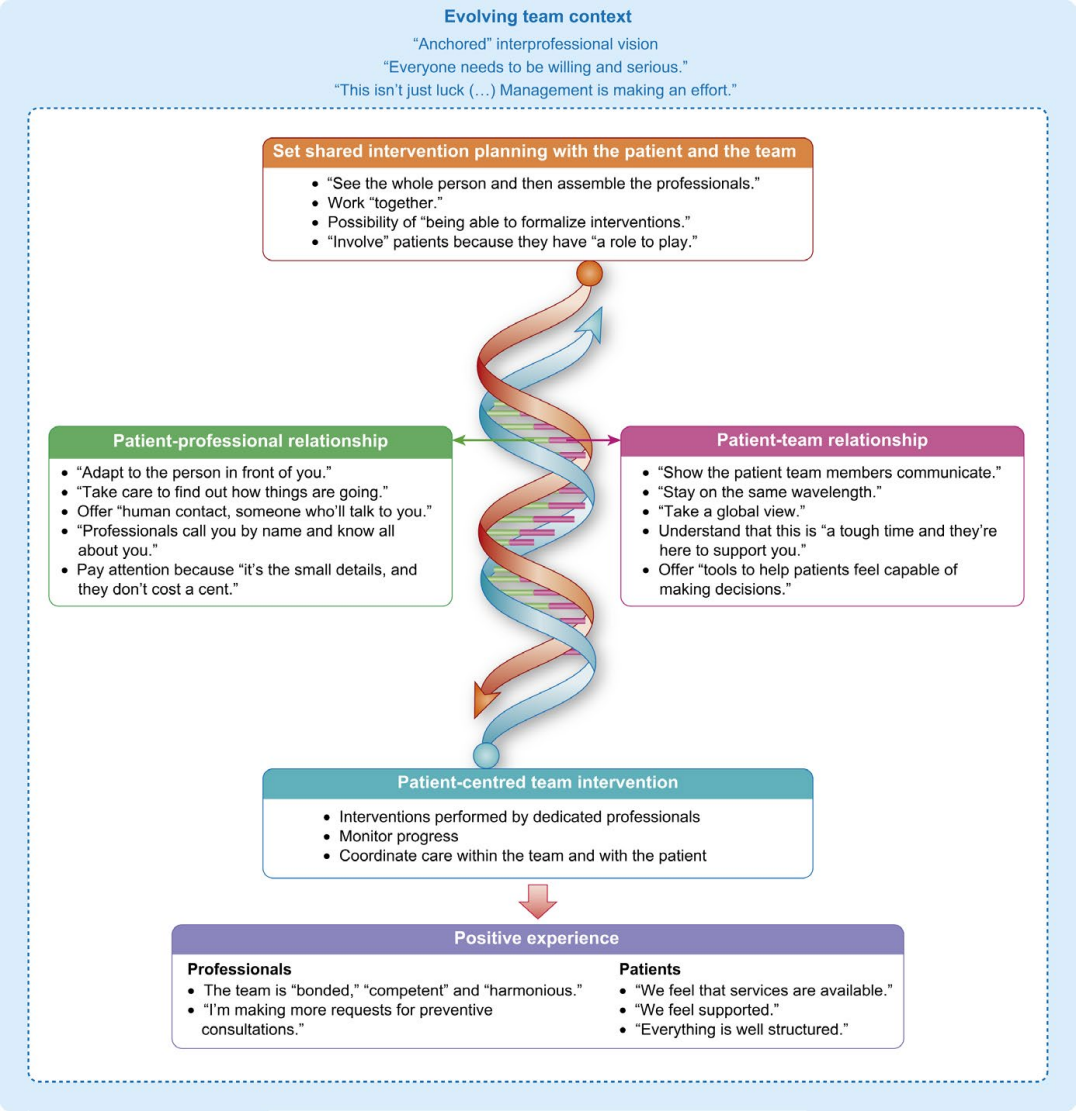
An alternative perspective of interprofessional patient‐centred practice in an oncology setting

In the wake of hypotheses that arose from the discussion and the authors’ evolving thought process, an alternative perspective of interprofessional patient‐centred practice was proposed as a starting point for a new avenue of discussion about this phenomenon (Figure 1). It is based on the results obtained by the secondary analysis, as well as nuances between two types of relationships (patient‐professional and patient‐team) that are central to IPPC patient‐centred practice (Figure 1). The DNA metaphor was chosen as the best way to present interprofessional patient‐centred practice, describing how it contributes to a more positive experience of care for patients. Verbatim comments from this study were added to more precisely detail the context of oncology care. First, the central components of the model, *shared intervention with the patient and the team* and *patient‐centred team intervention*, are illustrated by the two intertwined strands that form the double helix. The two activities are reciprocal, changing with the evolving context of the team and influenced by the patient's, the professional's and the team's ability to be in a relationship. The paired bases represent the entwined relationships between the *patient and the professional* and *the patient and the team*, corresponding to the ability and characteristics of patients and professionals to be in a relationship. Interprofessional patient‐centred practice is constantly changing, due to the ongoing interaction between planning and carrying out the intervention, as well as the evolving context. This illustration suggests that by focusing on the relationship between the professionals and the patient, teams can readjust their interventions more quickly and efficiently to respond to needs identified by the patient and promote the exchange of information, as well as access to and continuity of care and services. An interprofessional patient‐centred practice is thus conducive to a more *positive experience* for professionals and patients alike.

### 4.2 Strengths and limitations

Empirical validation involves several challenges, including the challenge of successfully conceptualizing phenomena that are simultaneously complex, evolving and collective. Using secondary analysis, we were able to estimate the parameters for the use of the *IPPC Practice Framework* and its capacity for showing interprofessional patient‐centred practice in a “real‐life” context. One strength of this study was that data from typical cases represented the reality experienced by oncology teams in Quebec (Canada) who adopted a practice model that promotes interprofessional practice and patient‐centred care. The data used were relevant, as they presented a rich array of perspectives (from patients and professionals) and interactions, although they may have been tinged with a certain social desirability. However, we were not able to document all aspects of the IPPC practice with both perspectives utilizing the IPPC Practice Framework. Bear in mind that one limitation of secondary analysis is that the initial data were collected to answer other research questions, and the interview guides that were developed did not reflect the *IPPC Practice Framework*. Also, we do not have absolute certainly that the practices of teams studied were patient‐centred. Despite the preliminary analysis showing patient‐centred concerns of the teams, maybe the IPPC practice was not performed. It should also be noted that the results of the present study apply to a specific context—oncology. Therefore, these results should be used in a similar sphere of application, that is, in a public health system with patients who have received various cancer diagnoses.

## 5 CONCLUSION

The utilization of the *IPPC Practice Framework* enhanced our understanding of interprofessional patient‐centred practice from the practical and theoretical viewpoints. The theoretical framework enabled us to differentiate between the interprofessional practice of the three teams and structure the data that had been collected to develop processes and situate them in their context. However, it proved to be difficult to describe interactions among the various theoretical constructs to reveal the phenomenon. The analysis revealed that the representation of *patient‐centred processes*, the key component of the theoretical framework, was making it difficult to reflect the real practice of the teams. And so the question remains: “How does a team come to be patient‐centred?” or rather “How does a team come to be person‐centred?” One interesting avenue that could provide an answer to that question would be to consider person‐professional and person‐team relationships as being complementary, interrelated, and evolving, with multiple combinations being both possible and desirable, depending on the context. With that premise, we suggest an alternative perspective of IPPC practice to stimulate discussion about this subject. We believe that this representation fills a gap in the understanding of IPPC practice. Further studies will be needed to explore whether the proposed perspective is suitable for describing IPPC practice in a real‐life context. Moreover, IPPC practice needs to be deepened regarding key elements of patient‐centred care in oncology context as proposed by Cancer Care Ontario^12^ as well as the emergent approach of “patient as a partner”.^43^, ^44^ The professional skills inherent in such a practice should also be examined and integrated into the new model. The results of this study remind us that the study of the complex phenomena that are so closely tied to their context, such as interprofessional practice, requires us to use theoretical frameworks with caution. An empirical validation will need to be carried out to study the usefulness of the theoretical frameworks that have been created in order to refine our understanding of how a team can be now person‐centred.

## CONFLICT OF INTEREST

The authors report no conflict of interests. The authors alone are responsible for the writing and content of this article.
